# Eligibility for Liver Transplantation in Patients with Perihilar Cholangiocarcinoma

**DOI:** 10.1245/s10434-020-09001-8

**Published:** 2020-09-08

**Authors:** Jaynee J. A. Vugts, Marcia P. Gaspersz, Eva Roos, Lotte C. Franken, Pim B. Olthof, Robert J. S. Coelen, Jeroen L. A. van Vugt, Tim A. Labeur, Lieke Brouwer, Marc G. H. Besselink, Jan N. M. IJzermans, Sarwa Darwish Murad, Thomas M. van Gulik, Jeroen de Jonge, Wojciech G. Polak, Olivier R. C. Busch, Joris L. Erdmann, Bas Groot Koerkamp, Stefan Buettner

**Affiliations:** 1 Department of Surgery, Erasmus MC University Medical Center, Rotterdam, The Netherlands; 2 Department of Surgery, Cancer Center Amsterdam, Amsterdam UMC, University of Amsterdam, Amsterdam, The Netherlands; 3 Department of Gastroenterology, Maasstad Ziekenhuis, Rotterdam, The Netherlands; 4 Department of Gastroenterology and Hepatology, Erasmus MC University Medical Center, Rotterdam, The Netherlands

## Abstract

**Background:**

Liver transplantation (LT) has been performed in a select group of patients presenting with unresectable or primary sclerosing cholangitis (PSC)-associated perihilar cholangiocarcinoma (pCCA) in the Mayo Clinic with a reported 5-year overall survival (OS) of 53% on intention-to-treat analysis. The objective of this study was to estimate eligibility for LT in a cohort of pCCA patients in two tertiary referral centers.

**Methods:**

Patients diagnosed with pCCA between 2002 and 2014 were included from two tertiary referral centers in the Netherlands. The selection criteria used by the Mayo Clinic were retrospectively applied to determine the proportion of patients that would have been eligible for LT.

**Results:**

A total of 732 consecutive patients with pCCA were identified, of whom 24 (4%) had PSC-associated pCCA. Overall, 154 patients had resectable disease on imaging and 335 patients were ineligible for LT because of lymph node or distant metastases. An age limit of 70 years led to the exclusion of 50 patients who would otherwise be eligible for LT. After applying the Mayo Clinic criteria, only 34 patients (5%) were potentially eligible for LT. Median survival from diagnosis for these 34 patients was 13 months (95% CI 3–23).

**Conclusion:**

Only 5% of all patients presenting with pCCA were potentially eligible for LT under the Mayo criteria. Without transplantation, a median OS of about 1 year was observed.

Perihilar cholangiocarcinoma (pCCA) is the most common malignancy of the biliary tract and arises from biliary epithelial cells at the liver hilum.^1^^,^^2^ The annual incidence in Western countries is about 1–2 per 100,000.^3^ The standard curative option for patients with pCCA is a radical surgical resection which is associated with a median overall survival (OS) of 40 months after resection and a 5-year survival of 30–50%.^4^^,^^5^ The majority of patients with pCCA (> 80%), however, are considered unresectable at the time of presentation.^6^^,^^7^ The median survival of patients with unresectable disease is only 6 months, which can be prolonged to a median of 12 months by palliative chemotherapy including gemcitabine and cisplatinum.^6^–^8^

There are three main reasons for unresectability in patients with pCCA: the disease is locally advanced, metastatic disease is present, or patients are unfit for major surgery. Locally advanced disease is defined as invasion of surrounding organs or vasculature, or bilateral segmental biliary involvement, making surgical resection with negative resection margins and adequate liver remnant difficult.^9^ Previous studies have shown that surgical resection with a positive resection margin does not improve survival.^3^^,^^10^ Palliative resection in metastatic disease has not shown any survival benefit.^3^^,^^11^ Finally, resection of pCCA is high-risk surgical procedure with a postoperative 90-day mortality rate between 5 and 18% in Western series,^12^–^15^ and as such the possible benefit does not outweigh the risks in patients with advanced age, serious co-morbidity, or frailty.^16^–^18^

The Mayo Clinic and several other centers in the United States and Europe are currently treating a select subgroup of patients with locally advanced pCCA with neoadjuvant chemoradiation and liver transplantation (LT).^19^–^21^ In patients treated according to this protocol, a 5-year survival of 53%, slightly superior to the survival of patients after surgery for resectable disease, could be achieved.^19^^,^^20^ Even though these are excellent results, a pre-transplant dropout of 30% was noted, despite strict inclusion criteria that result in an extensive patient selection.^22^ These impressive results raise the question as to whether LT is underutilized for patients with pCCA. The first objective of this study was to apply the Mayo selection criteria to a consecutive cohort of pCCA patients to determine the eligibility rate for the Mayo Clinic LT protocol. The second objective was to compare outcomes of pCCA patients eligible for LT who underwent best supportive care or palliative chemotherapy with published outcomes of the Mayo LT protocol.

## Methods

### Data Collection

All patients with presumed pCCA between 2002 and 2014 at Erasmus MC University Medical Center, Rotterdam, and the Amsterdam UMC, Academic Medical Center, Amsterdam, the Netherlands, were identified by a systematic search in all medical records including: discharge letters, minutes of multidisciplinary hepatopancreatobiliary tumor board, radiology reports, operative reports, endoscopic reports, and pathology reports. Demographics (e.g., age and gender), clinical data [e.g., cholangitis, primary sclerosing cholangitis, body mass index (BMI)], and laboratory results [e.g., cancer antigen (CA) 19-9 levels] were collected from medical records. The Institutional Review Boards of both centers approved the study and the need for individual informed consent was waived.

pCCA was defined as a mass or malignant-appearing stricture at or near the biliary confluence, arising between the origin of the cystic duct and the segmental bile ducts.^2^ If no histopathological evidence was obtained, the diagnosis was established by the multidisciplinary hepatopancreatobiliary team based on clinical, radiological, endoscopic and laboratory findings, and follow-up.^23^

All imaging [i.e., contrast-enhanced CT and/or dynamic contrast-enhanced MRI or MRI with cholangiopancreatography (MRCP)] at the time of first presentation was revised by experienced abdominal radiologists. The radiologists were blinded for clinical information. Parameters assessed on imaging were tumor size, Bismuth–Corlette classification, lymph node and distant metastases, lobar atrophy, and vascular involvement. Vascular involvement was defined as apparent tumor contact of more than 180° to the portal vein or hepatic artery.^24^

Intrahepatic and extrahepatic metastases were defined as suspicion of metastases on imaging or found during staging laparoscopy or exploratory laparotomy. Lymph node metastases were classified in accordance with the AJCC staging manual 7th edition; i.e., lymph nodes beyond the hepatoduodenal ligament were classified as N2.

### Eligibility for Liver Transplantation

The inclusion criteria composed by the Mayo Clinic were applied to determine whether patients were eligible for LT.^20^ The inclusion and exclusion criteria are summarized in Table 1. The Mayo Clinic considered patients with pCCA to be those with positive brush/biopsy or malignant-appearing stricture or a mass on imaging with CA 19-9 > 100 U/ml. Patients had to be deemed unresectable (usually because of bilateral biliary involvement) or diagnosed with advanced liver disease due to primary sclerosing cholangitis (PSC). All patients should be medically fit for LT, interpreted here as WHO 0-2. As neoadjuvant chemotherapy for pCCA is not routinely prescribed for pCCA in the Netherlands, having received the full regimen could not be assessed as a selection criterion.

**Table 1:** Eligibility criteria. Mayo protocol for liver transplantation for pCCA. Patients should fulfill all criteria

Inclusion criteria	
1	Perihilar cholangiocarcinoma
2	Malignant appearing stricture on cholangiography; pathological confirmation; CA 19-9 > 100 U/ml; mass on imaging; polysomy on FISH
3	Unresectability or PSC diagnosis
4	Medical suitability for transplant
5	Completion of neoadjuvant chemotherapy
Exclusion criteria	
1	Intrahepatic/extrahepatic/lymph node metastases on imaging, or at staging laparoscopy/exploratory laparotomy
2	Prior malignancy < 5 years
3	Tumor > 3 cm anterior–posterior
4	Prior abdominal radiotherapy/resection of cholangiocarcinoma
5	Uncontrolled infection
Age criterion	
1	Age < 70 years

*CA 19*-*9* cancer antigen 19-9, *FISH* fluorescent in situ hybridization, *PSC* primary sclerosing cholangitis

Exclusion criteria of the Mayo protocol are intrahepatic, extrahepatic, or lymph node metastases, and a history of other malignancy within the last 5 years (excluding skin and cervical cancers). Lymph node metastases and distant metastases in our cohort were found at imaging, staging laparoscopy, or exploratory laparotomy. Suspicion based on imaging and suspicion/confirmation based on laparoscopy/laparotomy were pooled, as the clinical decision not to perform resection was based on both imaging and surgical exploration. Patients with tumor size over 3 cm of radial (i.e., anterior–posterior) diameter were excluded, as well as patients who had undergone prior surgery for pCCA or a transperitoneal biopsy of the tumor. Another exclusion criterion for LT was uncontrollable infection despite drainage procedures. Due to the retrospective nature of the database we could not apply this criterion to our patients, as clinical decisions pertaining to the uncontrollability of the infection could not be univocally reconstructed. Finally, patients aged older than 70 were not considered for LT in the Mayo Clinic.

An LT protocol for pCCA patients has existed in the Netherlands since 2011. The eligibility criteria are less strict than the Mayo protocol and include irresectability, no prior percutaneous drainage, tumor < 3 cm, and absence of lymph node and distant metastases.^25^ Patients do not receive neoadjuvant chemoradiation.

### Statistical Analyses

Statistical analyses were performed using SPSS version 24.0 (Armonk, NY). Overall survival (OS) was calculated from the date of first presentation in the tertiary referral center until death (event), last follow-up or loss to follow-up (censoring). Continuous data were reported as median with interquartile range (IQR). Categorical parameters were reported as counts and percentages. Survival was estimated using the Kaplan–Meier method and difference across groups was tested using the log-rank test.

## Results

**Fig. 1:**
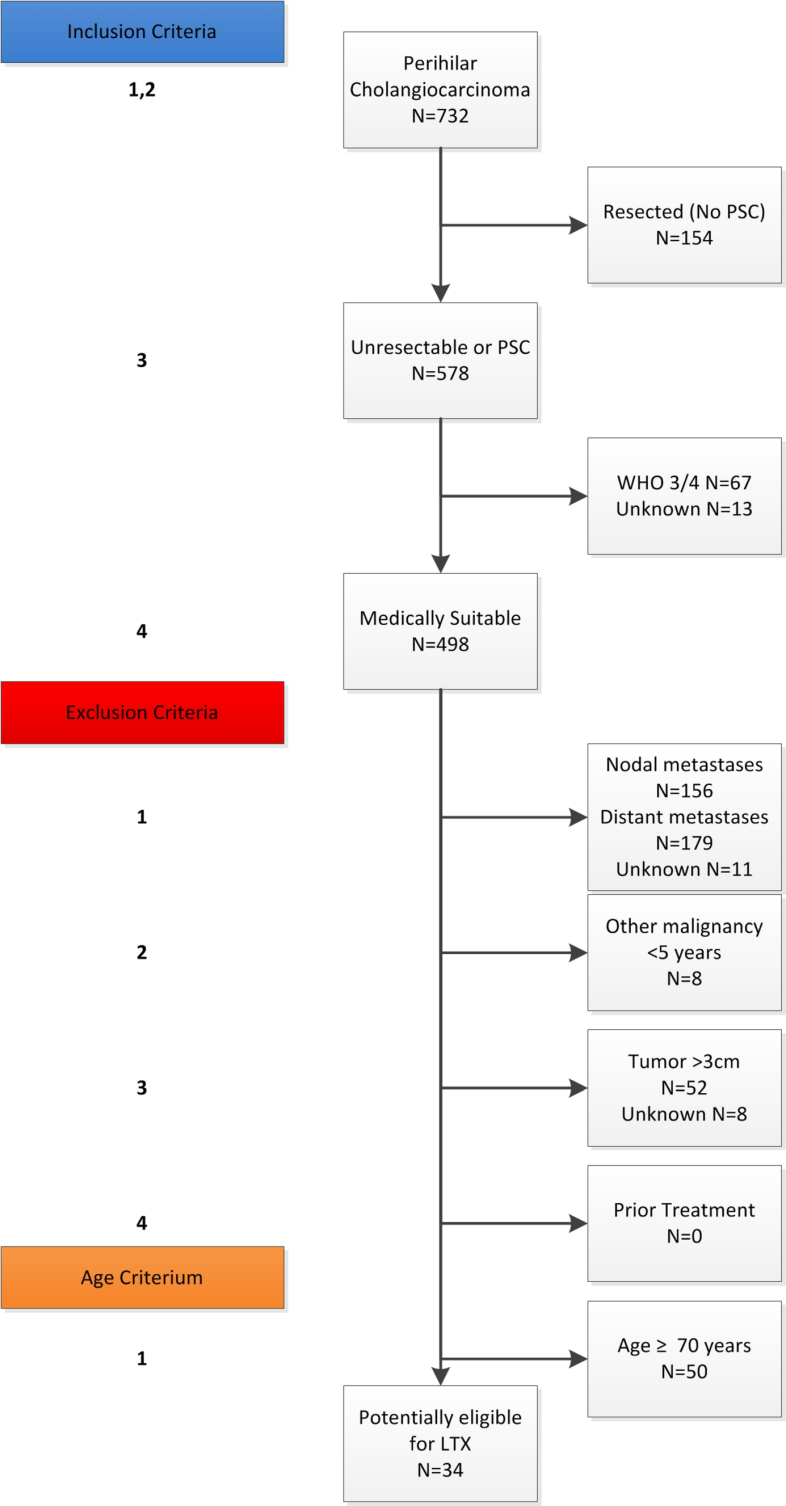
Liver transplantation eligibility. For nodal and distant metastases, suspicion based on imaging and suspicion/confirmation based on laparoscopy/laparotomy were pooled

A total of 732 consecutive patients with pCCA were identified (Table 2; Fig. 1). Of these patients, 462 (63%) were male with a median age of 67 years. Most patients (79%) had a good performance (WHO 0 or 1) status at the time of diagnosis, even though 297 (43%) had cholangitis before or at presentation in the referral center. Only 29 patients (4%) developed pCCA in the presence of PSC.

**Table 2:** Baseline characteristics

Characteristic	Whole cohort (*n* = 732)	Resected (*n* = 154)	Medically fit (*n* = 498)	Potential LT candidates (*n* = 34)
Age at first presentation, years	67 (58–73)	65 (55–72)	67 (58–74)	60 (54–65)
70 years or older	296 (40)	49 (32)	209 (42)	0 (0)
Gender, male	462 (63)	98 (64)	311 (62)	22 (65)
Year of presentation				
2001–2005	150 (21)	31 (20)	101 (20)	13 (38)
2006–2010	279 (38)	72 (47)	176 (35)	10 (29)
2011–2014	303 (41)	51 (33)	221 (44)	11 (32)
BMI, kg/m^2^	25 (22–27)	25 (22–27)	25 (22–27)	24 (22–28)
WHO performance status				
0	332 (46)	90 (59)	242 (49)	20 (59)
1	235 (33)	44 (29)	191 (38)	13 (38)
2	81 (11)	16 (11)	0 (0)	1 (3)
3	60 (8)	3 (2)	0 (0)	0 (0)
4	10 (1)	0 (0)	0 (0)	0 (0)
CA 19.9 (U/ml)^2^	215 (64–1278)	104 (34–343)	292 (87–1793)	160 (26–299)
≥ 1000 U/ml	98 (27)	7 (9)	82 (32)	1 (5)
Primary sclerosing cholangitis	29 (4)	0 (0)	27 (5)	2 (6)
Biliary drainage	681 (94)	141 (92)	462 (94)	31 (91)
Cholangitis before or at presentation in referral center	297 (43)	58 (38)	194 (42)	16 (52)

*BMI* body mass index, *WHO* World Health Organization, *CA 19*-*9* cancer antigen 19-9

On imaging, patients had a tumor with a median radial diameter of 2.7 cm (IQR 2.0–3.6; Table 3). Slightly more than half of the patients had macrovascular involvement on imaging. Suspected lymph node metastases were observed in 35% of the patients and about 1/3 of patients had Bismuth class IV biliary involvement (i.e., isolation of the second biliary radicle of both the left and right hepatic duct). Laparoscopic staging was performed in 210 patients (29%). Surgical exploration for resectable disease on imaging was conducted in 345 patients (47%), of whom 154 patients (44%) underwent a curative-intent resection (Fig. 1).

**Table 3:** Radiological staging and treatment

Characteristic	Whole cohort (*n* = 732)	Resected (*n* = 154)	Medically fit (*n* = 498)	Potential LT candidates (*n* = 34)
Tumor size, cm	2.7 (2.0–3.6)	2.1 (1.8–3.0)	2.9 (2.2–3.8)	2.3 (1.8–2.5)
Size > 3 cm	253 (38)	33 (22)	195 (44)	0 (0)
Vascular involvement				
PV involvement	376 (56)	58 (39)	277 (61)	17 (52)
Main/bilateral	133 (20)	6 (4)	103 (23)	11 (32)
HA involvement	366 (55)	47 (32)	279 (62)	15 (47)
Main/bilateral	90 (14)	3 (2)	74 (16)	3 (9)
Suspected lymph node involvement on imaging				
N1	192 (27)	31 (20)	141 (29)	0 (0)
N2	120 (17)	13 (9)	89 (19)	0 (0)
Distant metastases on imaging	76 (11)	5 (3)	62 (13)	0 (0)
Lobar atrophy on imaging				
None	514 (75)	107 (72)	348 (74)	27 (79)
Right	49 (7)	15 (10)	28 (6)	1 (3)
Left	127 (18)	27 (18)	94 (20)	6 (18)
Bismuth classification				
I	37 (6)	12 (8)	21 (5)	1 (3)
II	75 (11)	13 (9)	49 (11)	8 (24)
IIIA	180 (27)	47 (31)	114 (25)	7 (21)
IIIB	136 (20)	35 (23)	99 (22)	7 (21)
IV	235 (35)	34 (23)	172 (38)	11 (32)
Blumgart classification				
T1	204 (30)	69 (47)	117 (25)	9 (27)
T2	146 (22)	40 (27)	98 (21)	4 (12)
T3	326 (48)	39 (26)	245 (53)	21 (62)
Staging laparoscopy	210 (29)	83 (54)	118 (24)	8 (24)
Surgical exploration	345 (47)	154 (100)	178 (37)	19 (56)
Curative resection	160 (22)	154 (100)	6 (1)	2 (6)
Palliative surgery	13 (2)	0 (0)	11 (2)	1 (3)
LN metastases	115 (16)	0 (0)	70 (14)	(0)
Distant metastases	71 (10)	0 (0)	65 (13)	(0)
Systemic chemotherapy	56 (8)	5 (3)	49 (10)	2 (6)

*PV* portal vein, *HA* hepatic artery, *LN* lymph node

### Eligibility for Liver Transplantation

After exclusion of the patients who underwent resection, a further 80 patients were excluded based on WHO performance status (67 had WHO 3 or 4 and 13 patients had an unknown WHO status), leaving 498 patients that were medically fit to undergo LT (Fig. 1). These patients had similar baseline characteristics, yet worse tumor characteristics than the patients who underwent resection (Tables 1, 2). More patients in the unresected medically fit group had a tumor larger than 3 cm (44% vs. 22%; Table 3). The prevalence of invasion of portal vein (61% vs. 39%) and/or hepatic artery (62% vs. 32%) invasion was also higher in the medically fit group. Patients more frequently had Bismuth class IV disease (38% vs. 23%), and Blumgart T3 (53% vs. 23%). Finally, this group more often had suspected N2 nodes on imaging (19% vs. 9%) and more confirmed nodal metastases during (exploratory) surgery (14% vs. 0%). The same was the case for suspected (13% vs. 3%) and confirmed (13% vs. 0%) metastatic disease.

As part of the oncological exclusion criteria, 335 patients were excluded because of lymph node metastases (*n* = 156) or distant metastases (*n* = 179; Fig. 1). Nodal or distant metastases were diagnosed in 200 patients (59.7%) based on radiological imaging or staging laparoscopy, and in 135 (40.3%) patients during exploratory surgery. Six patients had another malignancy < 5 years before diagnosis, 1 patient had prostate cancer with palliative treatment, and 1 patient had breast cancer with palliative treatment. On imaging, 52 patients had a tumor larger than 3 cm, with a median tumor size of 3.7 cm (IQR 3.5–4.5). No patient received prior treatment for the tumor. Finally, 50 patients were older than 70 years and were excluded based on the age criterion only.

### Possible Liver Transplantation Candidates

After applying the Mayo Clinic inclusion and exclusion criteria, 34 patients were eligible out of a total cohort of 732 pCCA patients (5%; Fig. 1; Table 2). The median age was 60 years (IQR 54–65; Table 2) and 22 patients were male (65%). Two patients (6%) had PSC-associated pCCA. In most patients, one or more traditional contraindications for resection were present: main portal vein involvement was present in 11 patients (32%), while common hepatic artery involvement was observed in 3 patients (9%). Lobar atrophy was present in 7 patients (21%). One in three patients had Bismuth class IV disease (*n* = 11; 32%). Twenty-one patients had Blumgart T3 disease (62%). Most patients had surgically confirmed locally advanced disease: staging laparoscopy was performed in 8 patients (24%); while an exploratory laparotomy was performed in 19 patients (56%). Thirteen patients (38%) did not undergo either staging laparoscopy or exploratory laparotomy.

Eleven eligible patients were diagnosed after 2011, seven of whom underwent exploratory laparotomy. Two patients underwent an LT, making the percentage of patients who underwent LT in the 2011–2014 period 18% (i.e., 2 out of 11). One patient who had PSC-related pCCA received an LT in our cohort. Another patient was transplanted for Bismuth IV non-PSC related pCCA.

### Survival Estimates

**Fig. 2:**
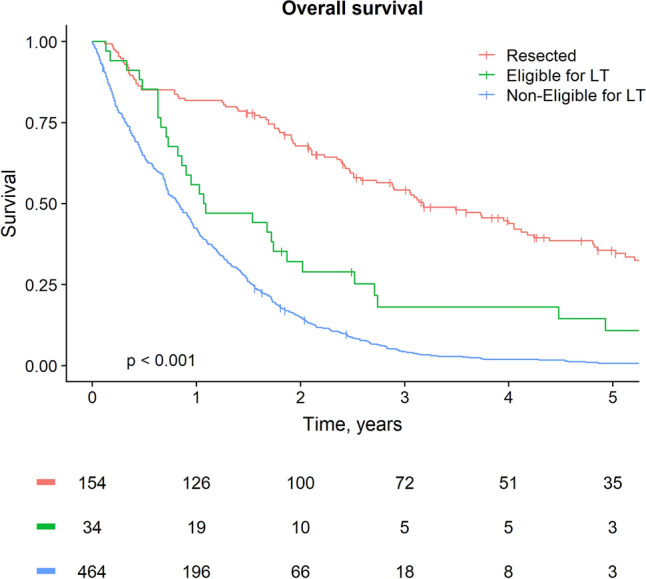
Overall survival

The median follow-up of patients alive at last follow-up was 4 years. During follow-up 661 patients (90.3%) died. Median OS after diagnosis was 12 months (95% CI 11–14). For patients who underwent a resection, the median OS was 38 months (95% CI 29–48; Fig. 2), and 1-, 3-, and 5-year survival rates were 82%, 54%, and 36% respectively. Median OS for patients eligible for LT was 13 months (95% CI 3–23; *p* < 0.001) without LT. The survival at 6 months was comparable between patients eligible for LT and resected patients (85% vs. 85%) and superior to the 6-month survival of ineligible patients (65%). The 1-, 3- and 5-year survival rates in the patients eligible for LT were 56%, 18%, and 11%.

The patient who underwent LT for PSC-related pCCA in our cohort is alive without recurrence at last follow-up, 4 years and 10 months after diagnosis. The patient with non-PSC related pCCA died of disease recurrence after 2 years and 11 months.

## Discussion

This study on the applicability of the Mayo Clinic LT protocol was conducted in a large consecutive cohort of 732 pCCA patients of whom only 34 (5%) would have been for LT. Most patients who were ineligible had poor performance status, nodal or metastatic disease, tumor size > 3 cm, age > 70 years, or a combination of these factors. Approximately 20% of patients are currently treated with curative resection. Only two of the eligible patients underwent LT according to the Dutch protocol, out of 11 patients diagnosed after its implementation in 2011. The majority of patients eligible for LT underwent best supportive care with a median OS of only 13 months and a 5-year OS of 11%, which is clearly inferior to OS reported for LT by the Mayo protocol. In patients treated according to this protocol, a 5-year OS of 53% for patients who started with neoadjuvant chemoradiation, and 70–90% 5-year OS after LT was observed.^19^^,^^20^^,^^26^ The survival at 6 months was comparable between patients eligible for LT and resected patients (85% vs. 85%), indicating that neoadjuvant treatment may be feasible in these patients.

The prognosis of unresectable pCCA is poor.^27^^,^^28^ In our study, even in the selected cohort of 34 patients that would have been eligible for LT, median survival was only slightly more than a year. In contrast, patients who underwent resection had a median survival of more than 3 years. This is a significant difference between patients who are, in oncological terms, not always very different. Therefore, LT may provide a treatment option for patients with locally advanced disease, who currently have limited treatment options. Indeed, the only treatment option available to these patients is systemic treatment with gemcitabine and cisplatinum.^8^ With LT, large steps can be made with regards to survival. A 2016 study based on the European Liver Transplant registry reported on 105 unresectable patients up until 2010. Of these patients, 6 (5.7%) had confirmed PSC-related pCCA, while 16 underwent neoadjuvant chemoradiation. After LT, a 5-year overall survival of 32% was observed.^29^

A striking difference between the Mayo Clinic cohort and our cohort is the number of patients diagnosed with PSC, an important risk factor for pCCA.^19^ In their largest study to date, 63% of patients had PSC in the Mayo Clinic cohort. The rate of patients with PSC in the other centers was also 63%.^20^ Even though epidemiological studies show the rate of PSC in pCCA patients might be as high as 10% in some regions, this is a large overrepresentation.^30^ In contrast, in our cohort only 4% of the total patient population was diagnosed with PSC. This rate was 6% in the patients that would have been eligible for LT. Because cancer surveillance of patients with PSC is advised, pCCA is possibly diagnosed at an earlier stage.^31^ In addition, patients with technically resectable disease and PSC were also eligible for LT. This has likely resulted in overrepresentation of PSC patients as well as a lower stage and better OS. This is also demonstrated by the superior 5-year per protocol recurrence-free survival of 72% in PSC-related pCCA, compared with 51% in non-PSC-related pCCA (*p* = 0.06) in the Mayo series.^20^ The difference in PSC rate possibly also explains the younger age (51 years vs. 60 years) and consequently better physical condition of patients in the Mayo Clinic cohort. More differences might exist between incidental pCCA and PSC-associated pCCA patients, in whom oncogenic mutations are sometimes noted before clinical manifestations and who have an aberrant DNA methylation profile.^32^–^34^ Clinical implications of these differences have yet to be elucidated.

In most countries performing LT, a shortage of donor livers exists. This is illustrated by the Eurotransplant 2017 report, with 2548 patients on the waiting list in 2017, and 1674 patients transplanted.^35^ A possible solution for this problem is living-donor liver transplantation (LDLT). After the first successful LDLT was performed by Strong and colleagues in Australia, there was an initial surge in LDLT in most Western countries.^36^^,^^37^ In the following years, however, most centers have abandoned the practice in both Europe and the US, because of concerns of donor safety and technical challenges.^37^ As LDLT increases the number of available donor livers, it might enable transplantation in patients with an expected 5-year survival that is lower than typically accepted for LT, such as pCCA patients.^37^^,^^38^ The 29% rate of living donors in the Mayo Clinic cohort is evidence that LDLT is already being successfully utilized for pCCA.

The Mayo protocol age criterion is perhaps too strict and resulted in the exclusion of 50 patients aged 70 years or older, who met all other criteria. Parallel to the general aging population in the west, the average age of donors and recipients of LT is increasing.^39^^,^^40^ Both in the United States and Europe, the proportion of patients aged 65 years and older has increased to 20% in recent years.^39^ During the last decade, numerous studies reported the negative impact of sarcopenia and frailty in LT candidates.^41^–^43^ Large-scale retrospective studies into the impact of clinical aging markers on postoperative outcomes are already available, and show clinically significant differences even when age is taken into account.^44^–^46^ Rates of recipients > 70 years old currently vary between 3% in the United States and 1% in Europe.^35^^,^^47^ With strict selection, the outcomes of elderly liver recipients is already similar to younger patients.^35^^,^^48^^,^^49^ Despite current reluctance, the increasingly advanced methods to determine physiological condition as well as these promising LT results might be a reason for more liberal interpretation of calendar age criteria in the future.

In order to be included in the Mayo Clinic cohort, no definite pathological confirmation was required. Instead, the authors relied on a combination of clinical factors, including positive biopsy, CA 19-9 and preoperative imaging, reasoning that pathological confirmation by transluminal brush cytology or intraluminal biopsy is often not possible.^50^ In a previous article by the same group, pCCA could be confirmed in half of the explants of patients in whom it could not be demonstrated preoperatively.^23^ Due to the general scarcity of donor livers and an increasing number patients in need of an LT, this might raise concern.^35^^,^^40^ However, when combining patients with preoperative pathologic proof, pathologic proof at explant, and/or confirmed recurrence, only 5% of patients remained in whom, despite strong clinical suspicion, pCCA could not be pathologically confirmed.^20^ Therefore, pathological proof of pCCA pre-transplant may not be such a major clinical problem.

This study has a number of limitations inherent to its retrospective nature. First, 13 of the 34 patients (38%) in our cohort that fulfilled all Mayo protocol criteria did not undergo either laparoscopy or laparotomy as is prescribed in the Mayo protocol. This might have resulted in understaging, because lymph node or distant metastases were only evaluated on imaging in these 13 patients. In addition, it was not possible to define and exclude uncontrolled infection. As we did not take this exclusion criterion into account, the number of eligible patients might be even fewer. Therefore, the actual percentage of eligible patients may be lower than 5%. Finally, due to missing values, 32 patients (4%) were excluded that might have been eligible. As these limitations have opposite effects, we believe our final estimate of 5% for the percentage of patients presenting with pCCA that were eligible for the Mayo LT protocol to be reasonably accurate.

In conclusion, only about 5% of patients presenting with pCCA in a tertiary European setting will be eligible for the Mayo LT protocol. With LT, some of these patients will have a 5-year OS of 53%, that is clearly superior to the median OS of 1 year for patients eligible for LT who only receive systemic chemotherapy or best supportive care.
